# Recent advances of tanshinone in regulating autophagy for medicinal research

**DOI:** 10.3389/fphar.2022.1059360

**Published:** 2023-01-12

**Authors:** Sha Wu, Kui Zhao, Jie Wang, Nannan Liu, Kaidi Nie, Luming Qi, Lina Xia

**Affiliations:** ^1^ School of Health Preservation and Rehabilitation, Chengdu University of Traditional Chinese Medicine, Chengdu, China; ^2^ State Administration of Traditional Chinese Medicine Key Laboratory of Traditional Chinese Medicine Regimen and Health, Chengdu University of Traditional Chinese Medicine, Chengdu, China; ^3^ Key Laboratory of Traditional Chinese Medicine Regimen and Health of Sichuan Province, Chengdu University of Traditional Chinese Medicine, Chengdu, China; ^4^ College of Materials Science and Engineering, Southwest Forestry University, Kunming, Yunnan, China

**Keywords:** tanshinone, Salvia miltiorrhiza, autophagy, human cancers, traditional Chinese medicine, medicinal research

## Abstract

Initially described as an ancient and highly conserved catabolic biofunction, autophagy plays a significant role in disease pathogenesis and progression. As the bioactive ingredient of *Salvia miltiorrhiza*, tanshinone has recently shown profound effects in alleviating and treating various diseases by regulating autophagy. However, compared to the remarkable achievements in the known pharmacological effects of this traditional Chinese medicine, there is a lack of a concise and comprehensive review deciphering the mechanism by which tanshinone regulates autophagy for medicinal research. In this context, we concisely review the advances of tanshinone in regulating autophagy for medicinal research, including human cancer, the nervous system, and cardiovascular diseases. The pharmacological effects of tanshinone targeting autophagy involve the regulation of autophagy-related proteins, such as Beclin-1, LC3-II, P62, ULK1, Bax, ATG3, ATG5, ATG7, ATG9, and ATG12; the regulation of the PI3K/Akt/mTOR, MEK/ERK/mTOR, Beclin-1-related, and AMPK-related signaling pathways; the accumulation of reactive oxygen species (ROS); and the activation of AMPK. Notably, we found that tanshinone played a dual role in human cancers in an autophagic manner, which may provide a new avenue for potential clinical application. In brief, these findings on autophagic tanshinone and its derivatives provide a new clue for expediting medicinal research related to tanshinone compounds and autophagy.

## Introduction

**FIGURE 1:**
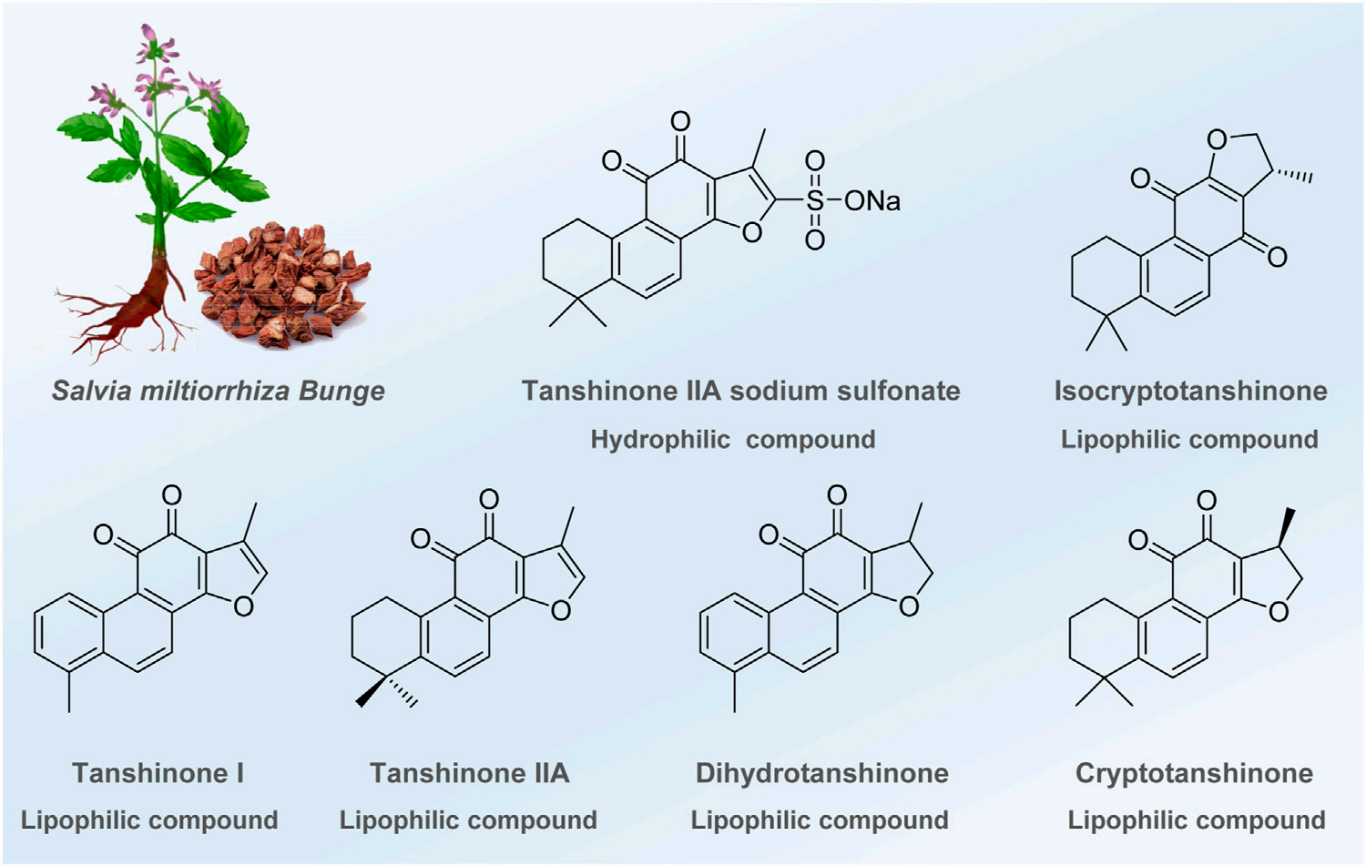
Bioactive ingredients identified or modified from *Salvia miltiorrhiza* in regulating autophagy.

In the long history of human society, over hundreds of thousands of years, traditional Chinese medicine (TCM) and botanical medicine have played significant roles in protecting human beings from diseases (Yuan et al., 2016; Caesar and Cech, 2019; Atanasov et al., 2021; Scherlach and Hertweck, 2021). Among them, *Salvia miltiorrhiza*, one of Asia’s most widely used medicinal herbs, demonstrates impressive clinical efficacy against various diseases (Su et al., 2015; Shaito et al., 2020; Ansari et al., 2021), such as cardiovascular and cerebrovascular diseases (Su et al., 2015; Li et al., 2018), liver cirrhosis (Shi et al., 2019), and chronic renal failure (Shao et al., 2021). With modern medicine’s rapid and remarkable development, the scientific community has gradually revealed the magical medicinal effect of *Salvia miltiorrhiza*. In recent years, multiple reviews have been conducted to decipher the mechanisms and found that anti-inflammation, anti-oxidation, anti-fibrosis, anti-apoptotic, etc., are the main pharmacological effects of this traditional Chinese medicine. Despite the remarkable achievements made above, recent experiments have proven that several tanshinones and their derivatives also play an essential role in regulating the autophagic process to cure different diseases. These potential molecules that regulate autophagy are divided into two types (Figure 1): a) hydrophilic components, which are water-soluble substances such as tanshinone IIA sodium sulfonate (TSN-SS), and b) extensively studied lipophilic components, such as tanshinone I (Tan I), tanshinone IIA (Tan IIA), dihydrotanshinone, and (iso) cryptotanshinone. Tan IIA, a lipophilic diterpene quinone, has positively affected various diseases, such as coronary heart disease, angina pectoris, and Alzheimer’s disease (Gao et al., 2012; Ren et al., 2019; Zhou et al., 2021). Experimental results prove that Tan IIA can regulate cell apoptosis, inhibit cell proliferation and migration, and promote cell viability to treat the diseases mentioned above (Ansari et al., 2021). TSN-SS, a water-soluble compound obtained by sulfonation of Tan IIA, has been approved by the China Food and Drug Administration (CFDA) to treat cardiovascular diseases (Zhou et al., 2019). Moreover, Tan I, another crucial bioactive component of *Salvia miltiorrhiza*, shows anticancer, neuroprotective, and anti-inflammatory activities (Chen et al., 2014; Li et al., 2018). These advancements provide an opportunity to discover new drugs from *Salvia miltiorrhiza*.

Autophagy, as an evolutionarily ancient and highly conserved catabolic process, is a self-protective mechanism of cells and plays an essential role in regulating cellular homeostasis under physiological and pathophysiological conditions (Mizushima, 2018; Yim and Mizushima, 2020). Generally, the autophagy process involves three steps: the formation of double-membrane vesicles, the formation of autophagosomes by phagocyte proteins and organelles, and subsequent fusion with lysosomes to complete the degradation process (Parzych and Klionsky, 2014). Under normal conditions, autophagy is conducive to maintaining cellular homeostasis, while defects in autophagy function can cause abnormal tissue function and are associated with various diseases. For example, dysregulated autophagy is involved in the pathogenesis of inflammatory bowel disease by producing proinflammatory cytokines and promoting intestinal epithelial cell stress (El-Khider and McDonald, 2016). The absence of autophagy pathways leads to the accumulation of two proteins, tau, and β-amyloid peptide, which are the main pathological changes in Alzheimer’s disease (Li et al., 2017). Recent studies have indicated that the regulation of autophagy plays a non-negligible role in treating diabetes mellitus (Kim and Lee, 2014; Zhang et al., 2018), Parkinson’s disease (Nixon, 2013; Tran and Reddy, 2021), chronic obstructive pulmonary (Racanelli et al., 2018), and malignant tumors (Galluzzi et al., 2017; Li et al., 2020). Of note, Lu et al., 2019 demonstrated that an autophagosome-tethering compound (ATTEC) specifically induced mutant huntingtin protein (mHTT) degradation by targeting pathogenic proteins to autophagosomes through interaction with autophagy-associated protein LC3 and the pathogenic protein mHTT of Huntington’s disease (HD). This result demonstrated the possibility of using autophagosome-coupled compounds to target protein degradation and provided a new opportunity for drug discovery (Li et al., 2019). Moreover, the treatment of hepatocellular carcinoma by artesunate through glucosylceramidase-mediated autophagic degradation has entered preclinical investigation (Chen et al., 2022). Hence, developing relevant drugs targeting autophagy will be a promising strategy for treating diseases that are unavailable with conventional medicinal treatment. Indeed, remarkable progress has been made in discovering small molecules targeting autophagy, such as chloroquine and hydroxychloroquine, in recent years (Limpert et al., 2018; Xiang et al., 2020; Xie et al., 2021). However, current achievements gradually fail to fulfill the vast and complex drug needs in the clinic. Therefore, enriching the toolbox of clinical medicines is becoming a priority for researchers in relevant fields.

Phytochemicals from medicinal herbs and plants have always been a vital source for discovering new drugs, and the successful application of TSN-SS has been confirmed (Newman and Cragg, 2020; Miethke et al., 2021). In recent years, advances in pharmacological research focusing on *Salvia miltiorrhiza,* mainly concentrating on various cancers (Fang et al., 2021) and cardiovascular system diseases (Li et al., 2018; Ren et al., 2019), have been successively reported and systematically summarized. Nevertheless, compared with these achievements, the progress of tanshinone that regulates autophagy for curing various diseases lacks a concise and systematic review despite scattered literature documented in several reviews (Bao et al., 2020; Subedi and Gaire, 2021). Considering the therapeutic efficacy of tanshinone in the clinic, we concisely and systematically reviewed the advances of tanshinone identified from *Salvia miltiorrhiza* in treating various diseases by regulating autophagy functions in different cells and animal models. For the convenience of readers, this review is divided into three parts according to the types of autophagy-related diseases, including human cancer, nervous system diseases, and cardiovascular diseases. Additionally, the summary and outlook on this promising field are described at the end of this review. Together, these findings on autophagic tanshinone and its derivatives provide a new clue for expediting medicinal research related to tanshinone and autophagy.

## Targeting autophagy for human cancers

Autophagy plays a dual role in the initiation and progression of human cancers (Li et al., 2020; Yun et al., 2021). On the one hand, by preventing the accumulation of damaged proteins and organelles, autophagy limits oncogenic signaling, oxidative stress, and chronic tissue damage to suppress cancer initiation (White et al., 2015). On the other hand, cancer cells can utilize autophagy-mediated recycling to meet the elevated metabolic demand of growth and proliferation (White et al., 2015). This section introduces the potential of tanshinone to target autophagy for various human cancers, which are classified by signaling pathways (Amaravadi et al., 2019).

### PI3K/Akt/mTOR signaling pathway

The PI3K/Akt/mTOR signaling pathway is a classical signaling pathway that regulates autophagy and mainly includes phosphatidylinositol-3-kinase (PI3K), the serine/threonine kinase Akt, known as protein kinase B (PKB), and mammalian target of rapamycin (mTOR) (Jung et al., 2010). In recent years, studies have revealed that inhibiting the PI3K/Akt/mTOR signaling pathway can induce autophagic cell death by up-regulating the level of autophagy, which is an effective means to end or inhibit the progression of cancer (Popova and Juecker, 2021).

Glioma seriously affects patients’ quality of life as a malignant intracranial tumor. Ding et al. (2017) demonstrated that Tan IIA could inhibit glioma cell proliferation, promote cell autophagy and apoptosis, and play an antitumor role by suppressing the PI3K/Akt/mTOR pathway and increasing the expression of the proteins MAP1LC3 (LC3B), Beclin-1, and Bcl2-associated X (Bax) in U251 cells. This result might provide a new effective targeted therapeutic strategy for glioma. As a highly malignant tumor, melanoma lacks specific drugs except for early surgical treatment. Liu’s group proposed a model in which Tan IIA reduced A375 cell proliferation by activating autophagy by blocking the PI3K/Akt/mTOR/p70S6K1 signaling pathway and increasing autophagy-related genes such as Beclin-1 and LC3-II (Li et al., 2017). Meanwhile, the author confirmed that Tan IIA could reduce melanoma A375-induced tumor volume and weight in the mouse model. This result may inspire the search for new anti-melanoma drugs derived from Tan IIA. As the dominant form of liver cancer, hepatocellular carcinoma (HCC) is a fatal disease with no effective treatment or positive prognosis. Yuan’s work demonstrated that cryptotanshinone (CPT) could simultaneously induce apoptosis and autophagy in Huh7 and MHCC97-H cells by regulating the PI3K/Akt/mTOR signaling pathway, leading to up-regulation of related proteins such as poly ADP-ribose polymerase (PARP), Bax, caspase-3, LC3-II, Beclin-1, and ATG5. In addition, CPT was found to inhibit the growth of Huh7 xenograft tumors, and is expected to be a potential candidate therapeutic agent for HCC treatment (Luo et al., 2020). Lung cancer is one of the most common and worst cancers worldwide, with a 5-year survival rate of 10%–20% (Neal et al., 2019). A recent report demonstrated that isocryptotanshinone (ICPT) could induce pro-death autophagy by down-regulating the expression of p-Akt and p-mTOR in A549 cells, thereby inhibiting cell proliferation and exerting anti-lung cancer effects. (Guo et al., 2016). Breast cancer has overtaken lung cancer as the most newly diagnosed cancer globally. While exploring the antitumor mechanism of Tan IIA, Zhang and coworkers found that it could induce cell cycle arrest and autophagy by inhibiting the activation of the PI3K/Akt/mTOR pathway in MCF-7 breast cancer cells (Lv et al., 2018). In addition, another active component, Tan I, also showed inhibitory effects in ovarian cancer. In 2020, a research report provided the first evidence that Tan I promoted autophagy by inhibiting the PI3K/AKT/mTOR pathway and up-regulating the autophagy-associated proteins Beclin-1, ATG7, and LC3-II in A2780 and ID-8 ovarian cancer cells, leading to the inhibition of tumor growth. These reports provided new insight into the anticancer mechanism of tanshinone (Zhou et al., 2020).

### Beclin-1-related signaling pathway

The Beclin-1 gene was the first mammalian autophagy gene found by yeast two-hybrid screening in 1998 by Beth Levine’s group (Liang Xiao et al., 1998). Beclin-1 is extremely important for autophagic vesicle enucleation at the beginning of autophagosome formation as a critical regulator of autophagy. Several studies have revealed that Tan IIA displays an antitumor effect *via* the Beclin-1-dependent pathway. Hu’s group explored the antitumor effect of Tan IIA on human osteosarcoma MG-63 cells and found that concentrations of Tan IIA (5 and 10 mg/L) induced autophagic cell death and eventually contributed to apoptosis by the production of excessive ROS (Ma et al., 2016). Oral squamous cell carcinoma is one of the most malignant and harmful tumors. Ji’s group found that Tan IIA induced cell death in an autophagy-dependent manner in SCC-9 cells in a multipronged manner by inducing the Beclin-1/Atg7/Atg12-Atg5 signaling pathway and suppressing the PI3K/Akt/mTOR pathway (Qiu et al., 2018). Additionally, the author found that Tan IIA inhibited the growth of solid tumors in severe combined immune deficiency (SCID) mice in a Beclin-1-dependent manner. In addition, Tan IIA can also be used as an adjuvant drug for oral cell carcinoma. Wang’s group reported that Tan IIA exerted a strong radiosensitizing effect on SCC090 cells compared with the simple drug or single radiation treatment due to enhanced autophagy with increased protein levels of Beclin-1, Atg5, and LC3-II, the three critical proteins involved in autophagy (Ding et al., 2016). The drug combination is becoming an effective strategy for cancer treatment, and He’s group demonstrated that Tan I could induce pro-survival autophagy *in vitro* and *in vivo* by increasing the expression of the Beclin-1/Vps34 complex after treatment with Tan I by inhibiting B-cell lymphoma-2 (Bcl-2) expression in gastric cancer. The report also showed that the combination of the autophagy inhibitor chloroquine and Tan I could inhibit tumor growth more efficiently than monotherapy, which might be considered an effective strategy for treating gastric cancer (Jing et al., 2016).

### Reactive oxygen species

ROS is a general term for a class of active oxygen-containing compounds produced by aerobic metabolism organisms, including superoxide anions, free radicals, and peroxides (Rahal et al., 2014). Autophagy is one of the essential mechanisms for coping with ROS-mediated oxidative stress (Filomeni et al., 2015; Wu et al., 2022a). On the one hand, autophagy can be triggered to remove oxidized proteins or damaged organelles, thus maintaining cell homeostasis. On the other hand, when ROS accumulation is excessive or prolonged, autophagic death is subsequently induced. Therefore, the regulation of ROS and autophagy is significant for maintaining body homeostasis.

Cell death is an essential physiological process and regulating the autophagy of tumor cells is a good strategy for cancer therapy. Li and coworkers demonstrated that Tan IIA induced apoptosis and autophagy by accumulating ROS in PC-3 cells, thus inhibiting cancer cell growth and inducing cell death (Li et al., 2016). Notably, compared with the cells treated with Tan IIA, the combination of Tan IIA with 3-methyladenine (3-MA) increased the apoptotic ratio, implying that Tan IIA-induced apoptosis was mediated through autophagy in PC-3 prostate cancer cells. In addition, it has been reported that estrogen (ER) stress is closely related to cancer cell death and is an important downstream target of ROS. Guo’s group evaluated the anticancer potential of Tan I against glioblastoma and demonstrated that Tan I inhibited the proliferation of U87MG cells *via* the induction of apoptosis and G2/M cell cycle arrest (Jian et al., 2020). Mechanistic experiments indicated that Tan I triggered ER oxidative stress and Akt-mediated apoptosis by inducing the accumulation of intracellular ROS. Interestingly, protective autophagy was also triggered by the mechanism described above, supported by pretreatment with 3-MA, which effectively enhanced the Tan I-induced inhibition of U87 MG cells. These findings indicated that Tan I could be a potential anticancer drug candidate for glioblastoma treatment. For the multidrug-resistant colon cancer cell line SW620 Ad300, another report demonstrated that CPT induced autophagic cell death *via* the ROS-p38 MAPK-NF-κB signaling pathway. Importantly, their results also demonstrated a complementary relationship between CPT-induced apoptosis and autophagic cell death. This study indicated that CPT might be a potential chemotherapeutic agent for cancer treatment (Xu et al., 2017). In addition to the monomeric component, the mixed extract of total tanshinone (TDT) also plays a vital role in cancer treatment. Chen’s group found that TDT inhibited the proliferation of cancer cells by inducing apoptosis and protective autophagy by increasing the formation of intracellular ROS in 95D lung cancer cells and showed better cytotoxic effects than Tan IIA (Gao et al., 2015).

### AMPK-related signaling pathway

Adenosine 5′-monophosphate (AMP)-activated protein kinase (AMPK), a trimer composed of α, β, and γ subunits, is a critical kinase regulating bioenergy metabolism (Yuan et al., 2020). It is mainly affected by the adenosine monophosphate/adenosine triphosphate (AMP/ATP) ratio and maintains metabolic balance by regulating the activity of downstream signals (Faubert et al., 2015). Moreover, AMPK plays a positive role in autophagy. Under starvation conditions, the AMP/ATP ratio is increased, and phosphorylation of AMPKα activates AMPK, which phosphorylates the tuberous sclerosis complex (TSC) and regulatory-associated protein of the mammalian target of rapamycin (RAPTOR), and then inhibits the mTORC1 for the regulation of autophagy (Wu et al., 2015). In addition, the activation of AMPK also phosphorylates unc-51-like kinase 1 (ULK1), promoting its activity and activating the autophagy process (Fan et al., 2015). Li’s group explored the effect and mechanism of Tan I on breast cancer and found that Tan I could induce autophagy by up-regulating the phosphorylation of AMPKα and its downstream ULK1, thus effectively inhibiting the proliferation of the breast cancer cell line MDA-MB-231 (Zheng et al., 2020). Furthermore, the conduction of APMK upstream signaling molecules can also affect the regulation of autophagy. Su’s group demonstrated that Tan IIA-induced recruitment of the sestrin 2 (SESN2) promoter through the hematopoietic progenitor kinase-germinal cell kinase-like kinase/stress-activated protein kinase/c-Jun N-terminal kinase (HGK/SAPK/JNK-Jun) signaling axis led to SESN2/AMPK-α activation, thereby inducing autophagy and inhibiting osteosarcoma growth (Yen et al., 2018). In addition, Park et al. reported that CPT robustly activated the AMPK signaling pathway, including liver kinase B1 (LKB1), p53, and TSC2, suppressing mTORC1 in HepG2 cells and inducing autophagic cell death. These data suggest that CPT deserves further investigation as a novel specific AMPK activator in cancer treatment (Park et al., 2014).

### Miscellaneous signaling pathway

Apart from the pathways described above, components of *Salvia miltiorrhiza* can also regulate autophagy through other pathways to exert antitumor activity, such as the caspase-dependent mitogen-activated protein kinase/extracellular regulated protein kinase/mammalian target of rapamycin (MEK/ERK/mTOR) pathway (Qian et al., 2020), and p53/damage-regulated autophagy modulator (p53/DRAM)-mediated autophagy (Liu and Liu, 2020). Colon cancer is a common malignancy of the digestive tract. Wu’s group reported that dihydrotanshinone I (DHTS) promoted cancer cell apoptosis by mediating caspase-dependent autophagy through mitochondria in the human colon cancer HCT116 cell line (Wang et al., 2015). Later, Huo’s group found that Tan IIA stimulated autophagy in the colon cancer cell lines SW480 and HT29 by activating the MEK/ERK/mTOR pathway, thus inhibiting the growth of colon cancer cells (Qian et al., 2020). In addition, a report proposed by Chen indicated that another bioactive ingredient, CPT, induced apoptosis of HCT116 cells through ER stress-mediated autophagy, suggesting that CPT could be a promising therapeutic candidate for colorectal cancer treatment (Fu et al., 2021). P62/sequestosome1 (P62/SQSTM1) is a multifunctional protein that plays an essential role in autophagy. Kim’s group reported that Tan I exhibited antitumor activity in mesothelioma cells, and the activation of JNK and inositol-requiring enzyme 1 (IRE1) was critically involved in Tan I-induced p62/SQSTM1-dependent autophagy (Lee et al., 2017). As a critical tumor suppressor gene, p53 can also participate in the regulation of autophagy. Liu’s group investigated Tan I-induced cell apoptosis by repressing p53/DRAM-mediated autophagy in human hepatocellular carcinoma HepG2 and Huh7 cells (Liu and Liu, 2020). These results suggested that the bioactive ingredients of *Salvia miltiorrhiza* could be promising lead compounds for curing cancers through autophagy function.

Notably, Tan IIA is also used in combination therapy for cancer. Su’s group reported that Tan IIA potentiated the efficacy of fluorouracil (5-FU) in colon cancer in SCID mouse model by down-regulating LC3-II protein expression. This outcome suggested that Tan IIA may be a promising strategy for adjuvant chemotherapy for colon cancer (Su, 2012). In addition, Shen’s group first revealed that cotreatment with doxorubicin and Tan IIA activated autophagic cell death in drug-resistant gastric cancer cells, indicating that Tan IIA was a valuable agent with the potential to treat drug-resistant gastric cancer in combination therapy (Xu et al., 2018).

## Targeting autophagy for nervous system disease

Recent studies have disclosed that Tan IIA and its derivatives show potential in treating Alzheimer’s disease, age-related macular degeneration (AMD), and cerebral ischemic stroke in an autophagy-dependent manner (Ansari et al., 2021; Subedi and Gaire, 2021). Alzheimer’s disease (AD), caused by the accumulation of β-amyloid protein (Aβ), is a neurodegenerative disease that seriously affects the function of neurons (Yan and Vassar, 2014; Fan et al., 2020; Ju and Tam, 2022). Neurons rely heavily on autophagy to degrade dysfunctional cytoplasmic components, protein aggregates, and damaged organelles within cells to maintain the homeostasis of protein metabolism (Scrivo et al., 2018). Hence, eliminating the neurotoxicity caused by Aβ is considered a potential strategy for treating AD. Wan’s group investigated the possible neuroprotective mechanism of Tan IIA on Aβ_25–35_-induced spatial memory impairment in mice (Zhu et al., 2017). The results indicated that Tan IIA inhibited autophagy in the hippocampus by up-regulating RACK1 to suppress Beclin1 protein expression and down-regulate the LC3-II/I ratio, thereby attenuating Aβ_25–35_-induced spatial memory impairment in mice. As a progressive and devastating neurodegenerative malady, age-related macular degeneration (AMD) contributes to blindness among elderly individuals globally (Mitchell et al., 2018; Fleckenstein et al., 2021). Oxidative stress injury in the retinal pigment epithelium (RPE) is the main factor in the occurrence of AMD (Kauppinen et al., 2016; Kaarniranta et al., 2020), which triggers the degradation of RPE in an autophagic manner. Huang’s group investigated the protective effect and mechanism of TSN-SS on ARPE-19 cells under oxidative stress (Han et al., 2018). Their report showed that TSN-SS activated the PI3K/AKT/mTOR pathway to inhibit autophagy and diminished the expression of the autophagic proteins Beclin-1, ATG3, ATG7, and ATG9 in ARPE-19 cells under oxidative stress.

Meanwhile, oxidative stress has been shown to severely impact the nervous system during a cerebral ischemic stroke by generating ROS that leads to mitochondrial dysfunction, DNA integrity loss, and protein misfolding (Shi et al., 2019), followed by autophagic degradation to remove these dysfunctional substances. Han’s group established a cerebral ischemia model *in vivo* with hippocampal neurons cultured by hypoxia-glucose deprivation-reperfusion (OGD/R) (Zhu et al., 2017). The author found that Tan IIA activated the PI3K/Akt/mTOR signaling pathway by increasing the expression of p-p85, p-Akt, and p-mTOR and repressing LC3-II expression, and subsequently inhibited autophagy level. As a result, Tan IIA reduced cell death and protected hippocampal neuronal cells from reactive oxygen species. However, it is unknown whether Tan IIA inhibits OGD/R-induced autophagy by inhibiting the PI3K/Akt/mTOR pathway in neural cells. Compared with Tan IIA promoting autophagy for down-regulating the PI3K/Akt/mTOR signaling pathway in glioma cells (Ding et al., 2017), this exciting phenomenon firmly pointed out the possibility that Tan IIA may undergo neuroprotective activities through other pathways. Supportive experiments need to be performed for further investigation. Subsequently, researchers observed that TSN-SS inhibited middle cerebral artery occlusion (MCAO)-induced autophagy by down-regulating associated proteins, such as LC3-II, Beclin-1, and Sirt 6, and significantly reducing the infarct volume and brain water content (Wang et al., 2020). These reports offer valuable information for preventing and treating nervous system disease by using Tan IIA or TSN-SS in regulating autophagy function.

## Targeting autophagy for cardiovascular disease

Cardiovascular diseases, also known as circulatory diseases, are mainly caused by the blockage of the heart and blood vessels, featuring high prevalence, high disability, and high mortality (Sun et al., 2020; Goswami et al., 2021). Several pharmacological studies have proven that Tan IIA and its derivatives can treat various cardiovascular diseases by regulating autophagy *via* AMPK, miR-375/KLF4, MAPK, and other signaling pathways. Atherosclerosis (AS) is one of the leading causes of many cardiovascular diseases, such as coronary heart disease (Libby, 2021). Recent research has revealed that macrophages, another type of autophagy, play a pivotal role in destabilizing atherosclerotic plaques through the lysosome system (Tabas and Bornfeldt, 2016). Jia’s group elucidated that Tan IIA enhanced autophagy by inhibiting the miR-375/KLF4 mediated pathway, resulting in M2 polarization of macrophages and attenuating atherosclerosis. Moreover, increasing evidence indicates that the abnormal proliferation of vascular smooth muscle cells (VSMCs) is involved in the pathogenesis of AS and other cardiovascular diseases (Basatemur et al., 2019). Wang’s group demonstrated for the first time that Tan IIA could inhibit Ang II-induced proliferation and autophagy of VSMCs by down-regulating the p38/MAPK signaling pathway, suggesting that Tan IIA may be a potential novel medication for the treatment of cardiovascular disease that is associated with VSMC dysfunction (Lu et al., 2019).

As the most common complication of myocardial infarction (Musher et al., 2019), heart failure (HF) is highly associated with cardiomyocyte apoptosis (Bravo-San Pedro et al., 2017). Recent studies reveal that autophagy plays a protective role in disposing defective proteins in heart failure (HF) initiated by proteotoxicity. Wang’s group investigated the protective mechanism of Tan IIA on HF and found that Tan IIA increased autophagy and inhibited cardiomyocyte apoptosis by activating the AMPK/mTOR-dependent signaling pathway and up-regulating the expression of LC3 and Beclin-1. As a result, Tan IIA led to the protection of cardiomyocytes and improved cardiac function (Zhang et al., 2019). In addition, maladaptive myocardial remodeling consistently contributes to the poor prognosis of patients following myocardial infarction (MI). Hinek’s group demonstrated that TSN-SS could enhance autophagy through multiple mechanisms, including the up-regulation of Bcl-2, increased ratio of LC3 lipidation (LC3-II/LC3-I), and decreased level of autophagy substrate p62 (Mao et al., 2019). Meanwhile, they found that inhibiting the mTOR/P70S6K and TGFβ1/Smad3 signaling pathways, triggered by AMPK and Sirt-1, significantly reduced pathologic cardiac remodeling. These studies provide insight into the pharmacological mechanism of Tan IIA and offer a potential novel therapeutic strategy for managing MI. Myocardial dysfunction is organ damage caused partly by sepsis (Hsieh et al., 2020), which then initiates the autophagy program to remove dysfunctional or harmful substances. High mobility group box 1 (HMGB1), a late mediator of lethal systemic inflammation, can trigger sepsis when it leaks into the bloodstream (Deng et al., 2022). Wang’s group found that TSN-SS effectively facilitated the internalization of exogenous HMGB1 *via* active transport through clathrin- and caveolin-dependent endocytosis to LC3-positive cytoplasmic vesicles, such as amphisomes, for subsequent degradation *via* lysosome-dependent autophagy (Zhang et al., 2012). This discovery suggested that facilitating the return of endocytic HMGB1 to phagocytes may hold potential for the treatment of inflammatory-induced myocardial dysfunction. Myocardial injury is also considered an essential pathological phenomenon caused by sepsis. Mao and coworkers demonstrated that TSN-SS enhanced autophagy *via* the AMPK/mTOR signaling pathway and decreased NLRP3 inflammasome activation by regulating proinflammatory cytokine secretion Table 1 (Chen et al., 2021).

**TABLE 1:** Detailed information on tanshinone in regulating autophagy for human cancers.

Disease		Compound	Chemical property	Model	Dose	Mechanisms	Effect	Ref.
Cancer	Breast cancer	Tan IIA	lipophilic component	MCF-7 cells/Female BALB/c-nu nude mice	0-20 μM 30 mg/kg	↓PI3K/Akt/mTOR signaling pathway ↑LC3-II, the percentage of cells in S and G2 phase	↓Tumor growth ↑Autophagy	Lv et al. (2018)
	Breast cancer	Tan I	lipophilic component	MDA-MB-231 cells	10 μM	↑AMPK signaling pathway, Beclin-1, LC3-II ↑ULK1	↓Cell viability ↑Autophagy	Zheng et al. (2020)
	Colon cancer	CPT	lipophilic component	SW620 Ad300 cells	10 mM	↑ROS, LC3-II, ROS-p38 MAPK-NF-κB signaling pathway	↑Cell death ↑Autophagy	Xu et al. (2017)
	Colon cancer	DHTS	lipophilic component	HCT116 cells/Male NOD/SCID mice	0-12.5 μM/ 10 mg/kg	↑caspase-3 ↑LC3-II ↓p62	↑Cell death ↓Tumor growth ↑Autophagy	Wang et al. (2015)
	Colon cancer	Tan IIA	lipophilic component	SW480 cells, HT29 cells	10 μM	↑MEK/ERK/mTOR signaling pathway ↑LC3, Beclin1, ATG7 ↓p62	↓Tumor growth ↑Autophagy	Qian et al. (2020)
	Colon cancer	CPT	lipophilic component	HCT116 cells/zebrafishes	10 Μm/0-100 nM	↑caspase-3, LC3B, Beclin-1	↑Cell death ↑Autophagy	Fu et al. (2021)
	Colon cancer	Tan IIA	lipophilic component	Colo205 Colo205 colon cancer cells Male nude SCID mice	20 mg/kg	↓LC3-II	↑The efficacy of 5-FU ↓Autophagy	Su, (2012)
	Glioma	Tan IIA	lipophilic component	Glioma cells U251	100 ng/mL	↓PI3K/Akt/mTOR signaling pathway, Bcl-2 ↑LC3-II, Beclin-1, Bax	↑Cell death ↑Autophagy	Ding et al. (2017)
	Gliobla-stoma	Tan I	lipophilic component	U87 MG cells	0.625-10 μM	↑ROS, Bax, cleaved PARP, p21 ↓pAkt, Bcl-2, cyclin B1	↑Cell death ↓Cell proliferation cell cycle arrest ↑Autophagy	Jian et al. (2020)
	Gastric cancer	Tan I	lipophilic component	BGC823 and SGC7901 cells/Nude mice	0-40 μg/mL 20 mg/kg	↑Beclin-1/Vps34 complex, ↑LC3-II ↓Bcl-2	↓Tumor growth ↑Autophagy	Jing et al. (2016)
	Gastric cancer	Tan IIA	lipophilic component	SNU-719 and SNU-601 cells	5 μM	↑LC3-II, ↓p62	↑The anticancer effect of doxorubicin ↑Cell death ↑Autophagy	Xu et al. (2018)
	Hepatocellular carcinoma	CPT	lipophilic component	Huh7 and MHCC97-H cells Male Balb/c nude mice	12 µM 50 mg/kg	↓PI3K/Akt/mTOR signaling pathway, p62/SQSTM1, Bcl-2 ↑LC3-II, Beclin-1, ATG5	↑Cell death ↑Autophagy	Luo et al. (2020b)
	Hepatoma	CPT	lipophilic component	HepG2 cells Male BALB/c nu/nu mice	10 μM 2.5 mg/kg	↑LKB1, p53, TSC2, p-AMPK ↓m-TOR	↑Cell death ↑Autophagy	Park et al. (2014)
	Hepatoce-llular carcinoma	Tan I	lipophilic component	HepG2 and Huh7 cells	0-10 μM	↓p53 and DRAM ↓LC3-II, Beclin1	↑Cell death ↓Autophagy	Liu and Liu, (2020)
	Lung cancer	ICPT	lipophilic component	A549 cells	10 µM	↓p-Akt, p-mTOR ↑LC3-II, vacuoles	↓Tumor growth ↑Autophagy	Guo et al. (2016)
	Lung Cancer	TDT	lipophilic component	95D Cells	0-8 μg/mL	↑LC3-II, Beclin-1, Atg3, Atg5, Atg7, and Atg12, ROS, caspase-3, cleaved PARP	↑Cell death ↑Autophagy	Gao et al. (2015)
	Malignant melanoma	Tan IIA	lipophilic component	A375 cells/mice	0-4.0 μg/mL 50 μg/g	↓PI3K/Akt/mTOR/p70S6K1 signaling pathway ↑LC3-II ↑Beclin-1	↓Tumor growth ↑Autophagy	Li et al. (2017b)
	Malignant pleural mesothe-lioma	Tan I	lipophilic component	H28 cells	0-40 μM	↑IRE1, CHOP and p-JNK ↑p62/SQSTM1 and LC3-II	↑Cytotoxicity ↑Autophagy	Lee et al. (2017)
	Ovarian cancer	Tan I	lipophilic component	Human A2780, mouse ID-8 cells/Female BALB/c nude mice	0-9.6 μg/mL 30 mg/kg	↓PI3K/Akt/mTOR signaling pathway, p62 ↑LC3-II ↑Beclin-1 ↑Atg7	↓Tumor growth ↑Autophagy	Zhou et al. (2020)
	Osteosarcoma	Tan IIA	lipophilic component	MG-63 cells	5-10 mg/L	↑Beclin-1 ↑LC3-II	↑Cell death ↑Autophagy	Ma et al. (2016)
	Oral squamous cell carcinoma	Tan IIA	lipophilic component	SCC-9 cells/BALB/c-nu mice	0-64 μM 0-35.08 mol/L	↑Beclin-1/Atg7/Atg12-Atg5 signaling pathway, LC3-II ↓PI3K/Akt/mTOR signaling pathway	↑Cell death ↓Tumor growth ↑Autophagy	Qiu et al. (2018)
	Oral squamous cell carcinoma	Tan IIA	lipophilic component	SCC090 cells	0-20 μM	↑Beclin 1, ↑Atg5, ↑LC3-II, ↑ROS	↑Radio-sensitizing ↑Cell death ↑Autophagy	Ding et al. (2016)
	Osteosarcoma	Tan IIA	lipophilic component	143B cells, MG63 cells/Male NOD/SCID mice	0-20 μM 20 mg/kg	↑HGK-SAPK/JNK-Jun signaling pathway ↑SESN2/AMPK-α signaling pathway, ↑Beclin-1, ATG5, ATG7, class III PI3K, ↓Bcl-2	↓Tumor growth ↑Autophagy	Yen et al. (2018)
	Prostate cancer	Tan IIA	lipophilic component	PC-3 cells	5 μM	↑Beclin-1, LC3-II, ROS, Bax/Bcl-2	↑Cell death ↑Autophagy	Li et al. (2016)

### Summary and outlook

**FIGURE 2:**
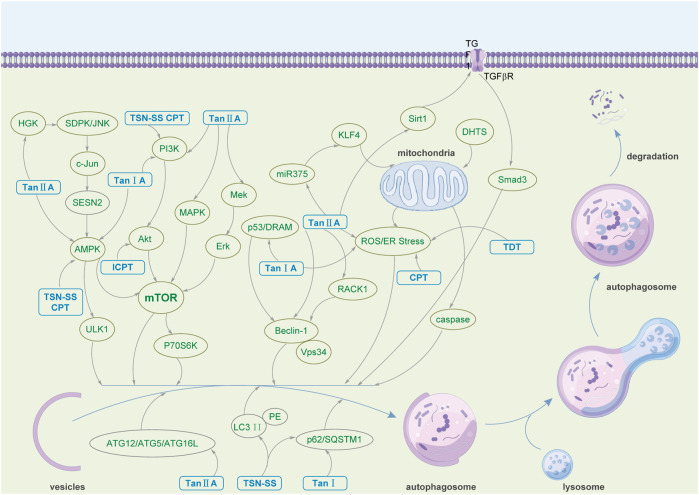
The pharmacological mechanism by which tanshinone regulates autophagy.

As an ancient and conserved cellular self-regulating biological function, autophagy plays a significant role in disease pathogenesis and progression. As the bioactive ingredient of *Salvia miltiorrhiza*, one of Asia’s most widely used traditional Chinese medicines, tanshinone has shown a profound effect in alleviating and treating various diseases by regulating autophagy. Table 2 summarizes that tanshinone demonstrates therapeutic effects on human cancer, the nervous system, and cardiovascular diseases. The mechanisms by which tanshinone participates in treating various diseases are diverse and complex. The multiple pharmacological effects of tanshinone mentioned above mainly involve the expression of autophagy-related proteins, such as Beclin-1, LC3-II, P62, ULK1, Bax, ATG3, ATG5, ATG7, ATG9, and ATG12; the regulation of the PI3K/Akt/mTOR, MEK/ERK/mTOR, Beclin-1-related, and AMPK-related signaling pathways; the accumulation of ROS and the activation of AMPK (Figure 2). As a promising drug candidate in cancer treatment, tanshinone exerts corresponding curative effects by promoting tumor cell death and inhibiting tumor cell proliferation. At the same time, it can also be used as a combination or adjuvant drug in cancer treatment, such as enhancing the efficacy of 5-FU and radiosensitization (Su, 2012). Moreover, Tan IIA and TSN-SS mainly exert neuroprotective activities by regulating the PI3K/AKT/mTOR signaling pathway and reducing the expression of autophagy-related proteins to inhibit autophagy functions. Additionally, Tan IIA and TSN-SS show potential in treating cardiovascular diseases by regulating autophagy, inhibiting the proliferation of VSMCs, reducing myocardial pathological remodeling, improving cardiac function, etc.

**TABLE 2:** Detailed information on tanshinone in regulating autophagy in the nervous system and cardiovascular diseases.

Disease		Compound	Chemical property	Model	Dose	Mechanisms	Effect	Ref.
Nervous system disease	Alzheimer's disease	Tan IIA	lipophilic component	Male Kunming mice	80 mg/kg	↑RACK1 ↓Beclin-1, LC3-II/I ratio	↓Neuronal damage ↓Autophagy	Zhu et al. (2017a)
	Age-related macular degeneration	TSN-SS	hydrophilic component	ARPE-19 cells	10 μM	↑PI3K/AKT/mTOR signaling pathway ↓Beclin 1, ATG3, ATG7, and ATG9	↓Cell death ↓Autophagy	Han et al. (2018)
	Stroke	Tan IIA	lipophilic component	HT-22 cells	2 μg/mL	↑Possibly by PI3K/Akt/mTOR signaling pathway ↓LC3-II	↓Cell death ↓Autophagy	Zhu et al. (2017b)
	Stroke	TSN-SS	hydrophilic component	Male wild-type C57BL/6J mice	40 mg/kg	↓LC3-II, Beclin-1, and Sirt 6	↓Brain edema and infarction ↓Autophagy	Wang et al. (2020)
Cardiovascular system disease	Atherosclerosis	Tan IIA	lipophilic component	Male wild-type C57BL/6J mice	10 mg/kg	↓miR-375 ↑KLF4	↑Cholesterol clearance repairing efferocytosis and M2 phenotype ↑Autophagy	Chen et al. (2019)
	Atherosclerosis	Tan IIA	lipophilic component	vascular smooth muscle cells	1-10 μg/mL	↓p38/MAPK signaling pathway ↑LC3-II, Beclin-1	↓Proliferation of VSMCs ↑Autophagy	Lu et al. (2019a)
	Myocardial infarction	Tan IIA	lipophilic component	H9C2 cells/mice	1 mΜ 1.5 mg/kg	↑AMPKs/mTOR signaling pathway, LC3-II, Beclin-1 ↓p62	↓Cell death ↑Cardiac function, cell viability ↑Autophagy	Zhang et al. (2019)
	Cardiomyopathy	TSN-SS	hydrophilic component	Male wild-type C57BL/6 mice	10 mg/kg	↑AMPK/mTOR signaling pathway, LC3-II, ATG5 ↓NLRP3 inflammasome, P62/SQSTM1	↓Myocardial inflammatory injury ↑Autophagy	Chen et al. (2021)
	Myocardial infarction	TSN-SS	hydrophilic component	Male wild-type C57BL/6J mice	10 mg/kg	↑LC3-II/I, Bcl-2, Sirt1, AMPK ↓p62, TGF-β/Smad3, mTOR/P70 S6K	↓Pathologic remodeling, cell death ↑Cardiac function ↑Autophagy	Mao et al. (2019)
	Myocardial dysfunction	TSN-SS	hydrophilic component	RAW 264.7 cells, HepG2 cells	100 μM	↑HMGB1 uptake, LC3-II	↓Proinflammatory mediators ↑Autophagy	Zhang et al. (2012)

According to the literature documented in this review, we are pleased to find that tanshinone has shown pharmacological effects on autophagy-related diseases. Current results have proven that using tanshinone in autophagy as a potential anticancer drug, especially Tan IIA, can be a potential strategy in the clinic. Continuous investments in tanshinone, including sufficient *in vivo* experiments and further pharmacological research, can benefit the discovery of novel drugs from *Salvia miltiorrhiza*. In addition, *Salvia miltiorrhiza* has been used for treating cardiovascular diseases throughout history, and remarkable curative effects concerning its bioactive ingredients have been investigated. Nevertheless, only a few reports have focused on the relationship between cardiovascular diseases and autophagy, suggesting great value and opportunity exist in this promising direction. Notably, we found that autophagy plays a dule role in cancer treatment. On the one hand, the regulation of autophagy by utilizing tanshinone can promote the inhibition of cancer cell growth and death by synergistic effects with apoptosis and cycle arrest. On the other hand, tanshinone promotes cancer cell survival by inducing protective autophagy that antagonizes the effects of apoptosis and other mechanisms. (Gao et al., 2015; Jing et al., 2016; Li et al., 2016; Luo et al., 2020; Jian et al., 2020). Therefore, disrupting the protective autophagy of tanshinone to enhance the anticancer effect is a possible therapeutic strategy, such as using autophagy inhibitors. Interestingly, compared with the monomeric component, total tanshinone shows better efficacy in alleviating cancers, suggesting that a synergistic effect of multiple tanshinone components may play a significant but unknown role in these biological processes (Gao et al., 2015). Sufficient experiments are needed to elucidate its potential mechanism, which is probably related to autophagy.

Moreover, some PI3K and mTOR inhibitors are currently used in antitumor research. Studies have shown that PI3K is widely overactivated in cancer and immune dysregulation and is closely associated with tumorigenesis and progression (Elmenier et al., 2019; Castel et al., 2021). Several PI3K inhibitors, such as buparlisib, copanlisib, alpelisib, duvelisib, and idelalisib, have been shown to produce clinically meaningful benefits in solid tumors and hematologic malignancies, and additional indications and combinations are being investigated (Furman et al., 2014; Baselga et al., 2017; Dreyling et al., 2017; Flinn et al., 2018; Juric et al., 2018; Juric et al., 2019). However, undesirable adverse drug reactions such as rash, hyperglycemia, diarrhea, and nerve damage have negated the clinical value of numerous candidate agents (Geuna et al., 2015; Lampson and Brown, 2017; André et al., 2019). In addition, mTOR is involved in regulating energy and metabolism (Hua et al., 2019). Inhibiting the mTOR pathway and thus curbing tumor growth is one of the classical antitumor strategies (Teng et al., 2019; Zou et al., 2020). However, the negative feedback mechanism of S6 kinase β-1 in this pathway makes the inhibitor-resistant (Zou et al., 2020). Recent strategies have focused on developing dual PI3K and mTOR inhibitors, which nicely circumvent the feedback loop (Wu et al., 2022b). As a potent PI3K/mTOR inhibitor, the excessively rapid metabolism of PI-103 *in vivo* has hindered its further clinical development. Although these shortcomings have been overcome to some extent, the consequent decrease in efficacy is another hurdle (Luo et al., 2020). Therefore, oncology therapeutic options targeting PI3K and mTOR are still in urgent need of development. Small molecule compounds of natural origin with low toxicity and high-efficiency properties have been a vital source for developing anticancer drugs (Dutta et al., 2019; Talib et al., 2021). This review describes that tanshinone has shown promising efficacy in various cancers through the multifaceted modulation of PI3K and mTOR targets. Notably, little literature has been reported on tanshinone having significant toxic side effects. Therefore, tanshinone is expected to be a powerful addition to the field of developing novel small-molecule inhibitors for the treatment of cancer.

Druggability has always been an inevitable topic in drug discovery (Zhang and Tang, 2018). Tan IIA and other lipid-soluble components have poor water solubility and bioavailability, significantly limiting their utilization as ideal drug candidates. One promising direction is to improve their bioavailability using chemical modification and nano-delivery systems (Aminu et al., 2020). Toxicity is also a critical problem that restricts the clinical application of tanshinone. Although the toxic effects of tanshinone and its derivatives on normal tissues have rarely been reported, sufficient experimental data are needed to evaluate its safety. Moreover, many side effects, such as fever, pain, and anaphylactic shock, occurred after TSN-SS injection, but the detailed mechanism was far behind its clinical application. The relationship between the above symptoms and autophagy may provide an answer (Zhou et al., 2019). Subsequently, conducting extensive and in-depth toxicological research on tanshinone and its derivatives is urgent and necessary to maximize clinical efficacy and avoid safety problems. In brief, we hope this review will shed light on the pharmacological mechanism by which tanshinone regulates autophagy and provide an experimental basis and inspiration for future research on tanshinone and autophagy.
